# Gaps and inconsistencies in the current knowledge and implementation of biosafety and biosecurity practices for rickettsial pathogens

**DOI:** 10.1186/s12879-024-09151-0

**Published:** 2024-02-29

**Authors:** Stuart D. Blacksell, Khanh Kim Le, Artharee Rungrojn, Jantana Wongsantichon, John Stenos, Stephen R. Graves, Nicholas P.J. Day

**Affiliations:** 1 Mahidol-Oxford Tropical Research Medicine Unit, Faculty of Tropical Medicine, Mahidol University, 10400 Bangkok, Thailand; 2 Centre for Tropical Medicine and Global Health, Nuffield Department of Medicine, Department of Medicine Research Building, University of Oxford, Nuffield, Oxford, UK; 3 Australian Rickettsial Reference Laboratory, University Hospital Geelong, Geelong, VIC Australia

**Keywords:** *Rickettsia* spp., *Orientia* spp., *Orientia tsutsugamushi*, Biosafety, Scrub typhus, Spotted fever

## Abstract

**Introduction:**

*Rickettsia* spp. and *Orientia* spp. are the causes of neglected infections that can lead to severe febrile and systemic illnesses in humans. Implementing proper biosafety practices when handling these pathogens is crucial to ensure a safe and sustainable work environment. It is essential to assess the current knowledge and identify any potential gaps to develop effective measures that minimise the risk of exposure to these pathogens. By doing so, we can establish a comprehensive framework that promotes safety, mitigates hazards, and safeguards the well-being of personnel and the surrounding community.

**Methods and results:**

This review aimed to synthesise and determine the evidence base for biosafety precautions for *Rickettsia* spp. and *Orientia* spp. pathogens. Enhancing our understanding of the relative infectious risk associated with different strains of *Rickettsia* and *Orientia* spp. requires identifying the infectious dose of these pathogens that can cause human disease. The application of risk groups for *Rickettsia* and *Orientia* spp. is inconsistent across jurisdictions. There is also incomplete evidence regarding decontamination methods for these pathogens. With regards to *Orientia* spp. most of the available information is derived from experiments conducted with *Rickettsia* spp.

**Conclusions:**

*Rickettsia* and *Orientia* spp. are neglected diseases, as demonstrated by the lack of evidence-based and specific biosafety information about these pathogens. In the case of *Orientia* spp., most of the available information is derived from *Rickettsia* spp., which may not be appropriate and overstate the risks of working with this pathogen. The advent of effective antibiotic therapy and a better understanding of the true hazards and risks associated with pathogen manipulation should inform decisions, allowing a sustainable and safe work environment.

**Supplementary Information:**

The online version contains supplementary material available at 10.1186/s12879-024-09151-0.

## Significance and impact of the study

Biosafety and biosecurity requirements for rickettsial pathogens require re-evaluation, including a nuanced approach to the assignment of pathogen risk groups and biosafety levels and risk mitigation strategies that enable scientists to work without unnecessary restrictions while maintaining a safe work environment.

**Supplementary Information:**

The online version contains supplementary material available at 10.1186/s12879-024-09151-0.

## Introduction

The World Health Organization (WHO) released the fourth edition of the Laboratory Biosafety Manual (LBM4) in 2020 [1], which advocates for risk-based biosafety using established knowledge. The WHO LBM4 emphasizes risk-based biosafety to improve laboratory biological risk management, particularly in low-resource settings [2]. This review discusses the general characteristics, biosafety evidence, and other important information regarding *Rickettsia* and *Orientia* spp. pathogens. These pathogens cause scrub typhus, typhus group, and spotted fever group rickettsiosis. Our review also highlights gaps in the current evidence and regulatory inconsistencies and provides recommendations for sustainable risk-based biosafety practices while working with *Rickettsia* and *Orientia* spp. pathogens using the principles promoted by the WHO.

## General

### Characteristics

The family *Rickettsiaceae*, order *Rickettsiales*, class Alphaproteobacteria, and phylum *Proteobacteria* are obligate intracellular Gram-negative bacteria, including the *Rickettsia* and *Orientia* genera [3]. The largest genus, *Rickettsia*, is divided into three antigenic subgroups responsible for several highly virulent diseases, including spotted fever (SFG) and typhus groups (TG), and a transitional group (TRG) comprising *R. australis*, *R. felis* and *R. akari* [4, 5]. The *Orientia* genus comprises the scrub typhus group (STG). There are more than 30 species of SFG rickettsiae, including the prototype *R. rickettsii* (causing Rocky Mountain spotted fever or RMSF) and other prominent members, including *R. conorii* (causing Mediterranean spotted fever or MSF) and *R. honei* (causing Flinders Island spotted fever). Regarding the TG, the two members are *R. typhi*, the causative agent of murine typhus, and *R. prowazekii*, the cause of epidemic typhus [6, 7]. *Orientia tsutsugamushi*, the STG prototype, causes scrub typhus and has several distinct antigenic types, including Karp, Kato and Gilliam [8]; however, more than 20 types of *Orientia* spp. have been identified.

### Clinical aspects

#### Clinical disease

Scrub typhus, typhus group and spotted fever group rickettsiosis have similar signs and symptoms, including fever, headache, rash, and muscle pain [7]. An eschar, a key pathognomonic sign of STG and SFG rickettsiosis, may sometimes be present as a dark scab-like lesion and can be found at the site of the mite or tick bite [7].

#### Modes of transmission

*Rickettsia* and *Orientia* are naturally transmitted to humans through the bites or infectious secretions of ectoparasites [5, 7, 9], and human-to-human transmission does not occur naturally [10]. The SFG rickettsiae are transmitted by ticks [5, 11], and TG members, *R. prowazekii* (epidemic typhus) and *R. typhi* (murine or endemic typhus) are transmitted by the body louse (*Pediculus humanus humanus*) and rat flea (*Xenopsylla cheopis*), respectively [5]. *Orientia tsutsugamushi* is transmitted by the bite of larval stage Leptotrombidium mites, also known as chiggers, in the primary endemic areas [12, 13].. Candidatus Orientia chiloensis [16] has also been detected in trombiculid mites of *Herpetacarus*, *Quadraseta*, and *Paratrombicula* spp.in Chile [14]. Transitional group members, *R. australis*, *R. akari* and *R. felis*, are transmitted by ticks, mites and fleas, respectively [7].

#### Treatment

Rickettsial diseases can be effectively treated with the tetracycline class of antibiotics. Doxycycline is the first choice due to its high efficacy [5, 15]. However, in the late 1990s, there was suspicion of doxycycline-resistant strains of *O. tsutsugamushi* following the observation of increased fever clearance times in a small number of scrub typhus patients in Chiangrai, northern Thailand [16]. Subsequently, studies have demonstrated that doxycycline-resistant scrub typhus is a misconception, with treatment outcomes likely to be determined by other bacterial, host, and pharmacological factors [17]. Azithromycin may be considered an alternative for scrub typhus treatment [15] but not murine typhus [18].

### Laboratory biosafety

#### Risk group classification and biosafety levels

*Orientia tsutsugamushi* and most of *Rickettsia* spp. are classified as risk group (RG) 3 pathogens in the United States, United Kingdom, Australia, Singapore, Germany, Switzerland, Belgium, and the European Union [19, 20]. However, there are inconsistencies in the designation of RGs depending on the pathogen and the country. Table 1 summarizes global RG classifications for *O. tsutsugamushi* and selected *Rickettsia* spp.

**Table 1:** Summary of risk group designation for *Rickettsia* spp. and *Orientia* spp. based on the ABSA international database [20]

Pathogen	USA BMBL 6th edition [35] (2020)	Australia/ New Zealand AS/NZS 2243.3.2010 (2010) [38]	Belgium (2008) [58]	Canada (2023) [36]	European Union Directive 2000/54/EC (2020) [59]	Singapore (2023) [60]	Switzerland (2013) [40]	United Kingdom ACDP [37] (2023)
*O. tsutsugamushi*	3	Not stated	3	3	3	Not stated	3	3
*R. aeschlimannii*		3	3	2		FSPII	3	
*R. africae*		3	3	2	3	FSPII	3	
*R. akari*	3	3	3	2	3	FSPII	3	3
*R. australis*	3	3	3	2	3	FSPII	3	
*R. bellii*	2	3	3	1		FSPII	3	
*R. canadensis*	2	3	3	3	3	FSPII	3	3
*R. conorii*	3	3	3	2	3	FSPII	3	3
*R. felis*				2				
*R. heilongjiangensis*			3	2	3	FSPII		
*R. helvetica*		3	3	2		FSPII	3	
*R. honei*		3	3	2		FSPII	3	
*R. japonica*	3	3	3	3	3	FSPII	3	
*R. massiliae*	3	3	3	2		FSPII	3	
*R. montanensis*	2	3	3	3	2	FSPII	3	3
*R. parkeri*	2	3	3	2	3	FSPII	3	3
*R. prowazekii*	3	3	3	3	3	FSPII	3	3
*R. rhipicephali*	2	3		3		FSPII	3	
*R. rickettsii*	3	3	3	3	3	FSPII	3	3
*R. siberica*	3	3	3	3	3	FSPII	3	3
*R. slovaca*			3	2		FSPII	3	
*R. typhi*	3	3	3		3	FSPII	3	3

NIH– National Institutes of Health, USA

BMBL– Biosafety in Microbiology and Biomedical Laboratories, 6th ed

ACDP - Advisory Committee on Dangerous Pathogens, 5th ed

FSPII– First Schedule Part II

#### Infectious dose

The infectious dose required to cause human infection for *Rickettsia* and *Orientia* pathogens remains insufficiently understood; however, for some rickettsiae, there are results from animal and in vitro models that can provide useful information. Using modelling, the estimated infectious dose for *R. rickettsii* is 23 ID_50_ (50% infectious dose), with a 95% confidence interval of 1 to 89 organisms [21] having used results from studies in non-human primates [22–24], and humans [25] (summarized study results presented in Table ;2). In vitro and in vivo studies have also been performed for other *Rickettsia* spp. in animal and in vitro models also using results from previous studies for *R. prowazekii* [26] (Cairo 3 strain), *R.typhi* [26], *R. canada* (now known as *R. canadensis*) [27], *R. rickettsii* [28], *R. conorii* [28] and *R. sibirica* [29] (Table ;2). Infectious dose results demonstrated differences in the susceptibility of the animal species [30], with guinea pigs (GP) generally more susceptible than mice (M), with minimum ID_50_ of 393 and 15 (M) and 1 and 8 (GP) for *R. prowazekii* (Breinl and Cairo 3 strains); 2 (M) and 5 (GP) for *R. typhi*; 9.7 × 10^4^ (M) and 1.1 × 10^4^ (GP) for *R. canada*; 1.1 × 10^4^ (M) and 126 (GP) for *R. rickettsii* (Sheila Smith) and 1.6 × 10^4^ (M) and 21(GP) for *R. rickettsii* (R strain) [30] (Table ;2). The infectious dose for *Orientia* spp. has not been determined, however, mouse [31] and hybrid models with non-human primates [32] have been developed for vaccine assessment purposes. It has been shown that there are differences in the severity of illness caused by various strains of *O. tsutsugamushi* in animals [33, 34]..

**Table 2:** Summary of results of studies investigating minimum infectious doses of rickettsiae in animal and in vitro models

Minimum infectious dose	PFU^a^	ID_50_^a^				FD_50_^a^	PFU^b^		Organisms		
	Chicken embryo cells	L cell	Chicken embryo	Mouse	Guinea pig	Guinea pig	Rat	Mouse BALB/c	Non-human primates	Humans	Reference
*R. typhi* Wilmington	3	15	325	2	5	11					[30]
*R. prowazekii* Cairo 3	5	3	20	15	9	9					[30]
*R. prowazekii* Brenl	2	2	41	393	1	2					[30]
*R. canadensis*	4	138	4	9.7 × 10^4^	1.1 × 10^4^	2.7 × 10^6^					[30]
*R. rickettsii* Sheila Smith	7	15	315	$\ge$1.0 × 10^5^	126	126					[30]
*R. rickettsii* R	2	0.5	7	1.6 × 10^6^	21	21					[30]
*R. conorii* Malish	2	0.7	220	1,692	47	319					[30]
*R. siberica* 246	3	1	62	6,300	23	34					[30]
*R. typhi* Wilmington							0.38–1.33	0.11–0.75			[61]
*R. rickettsii*									1.5		[24]
*R. rickettsii* Sheila Smith									45		[23]
*R. rickettsii* Sheila Smith									450		[22]
*R. rickettsii* Sheila Smith										13	[25]

PFU - plaque forming units

ID_50_ − 50% infectious doses

FD_50_ − 50% fever dose per gram of yolk sac

^a^ Numbers of rickettsiae that constitute 1 plaque forming units (PFUs) or 1 50% effective dose in terms of cytopathic degeneration, infection, or fever

^b^ Will seroconvert 50% of the exposed mice

^c^ Minimum amount in organisms required to induce mortality or morbidity in at least one Rhesus monkey test group following aerosol exposure

#### Biocontainment and personal protective equipment (PPE)

When assessing the level of risk involved in handling rickettsial pathogens, it is imperative to take into account both the nature of the pathogen and the type of procedure that will be performed. Factors such as pathogenicity, virulence, and the likelihood of aerosolization must be considered. Additionally, the specific activity to be performed (e.g., serology, inoculation of specimens into culture, animal experiments, initial in vitro growth of an isolate, or large-scale production) must also be taken into consideration as the risk will vary depending on the activity. Guidance is provided on these matters by the US [35], Canada [36], UK [37], Australia [38], Singapore [39], Switzerland [40] and Thailand [41] regarding recommendations for biocontainment requirements and special practices and procedures when working with rickettsial pathogens (see Table 3 for full details). In these jurisdictions, the guidance recommends that non-propagative work with clinical specimens be performed in a Biosafety Level (BSL) 2 facility using BSL2 practices for NAATs and serology testing. Canada, the UK, Australia, and Singapore require all in vitro propagation of the rickettsial pathogens to be performed at BSL3 containment. In the US, procedures involving the in vitro propagation of rickettsial pathogens are mandated to use “BSL3 practices and containment equipment… for activities involving culture propagation or specimen preparation and propagation of clinical isolates known to contain or potentially containing *Rickettsia* spp. pathogenic to humans.”. However, somewhat confusingly, the US regulations also state, “BSL2 facilities with BSL3 practices are recommended for all manipulations of known or potentially infectious materials, including… and inoculation, incubation, and harvesting of embryonated eggs or cell cultures.” The US has specific guidance for working with *Orientia* spp. pathogens [35] in the BMBL 6th edition; however, locations such as Australia, where scrub typhus is endemic (northern Queensland, Northern Territory) does not have specific guidance [38]. In vivo animal work with rickettsial pathogens is normally confined to BSL-3. However, recently, a mouse model for acute lethal rickettsial disease, using *R. parkeri* Atlantic Rainforest strain and C3H/HeN mice, has been described with the advantage that this model can be studied in an animal BSL2 containment level [42].

**Table 3:** Biocontainment and mitigation regulations for *Rickettsia* and *Orientia* spp

		Laboratory studies		
Country	Regulations	Activity	Low-risk	Enhanced-risk
USA	BMBL 6th ed [35]	Clinical	“BSL-2 practices and facilities are recommended for nonpropagative laboratory procedures with inactivated samples, including serological and fluorescent antibody procedures, nucleic acid amplification, and for the staining of impression smears after fixation.”	“BSL-3 practices and containment equipment are recommended for activities involving culture propagation or specimen preparation and propagation of clinical isolates known to contain or potentially containing Rickettsia spp. pathogenic to humans.” “Laboratory work with *Rickettsia* spp. may be conducted in a BSL-2 facility with enhanced special practices including strict access control, competency, and adherence to BSL-3 practices. Laboratories should be locked and access to non-essential personnel should be prohibited. BSL-3 practices include, but are not limited to, appropriate personal protective equipment (e.g., rear-closing gowns, gloves, eye protection, and respiratory protection such as N95 respirators or PAPRs), use of BSCs when handling any open container with potentially infectious material, and primary containment, such as sealed centrifuge rotors and other means of containment outside the BSC. Disruption of infected cells or yolk sacs should be accomplished within the BSC using an enclosed chamber to minimize the potential for aerosols. If eggs are used for propagation, the site of inoculation should be sealed with an appropriate sealant prior to transfer to an incubator. BSL-2 facilities with BSL-3 practices are recommended for all manipulations of known or potentially infectious materials, including the necropsy of experimentally infected animals and trituration of their tissues, and inoculation, incubation, and harvesting of embryonated eggs or cell cultures. Use of sharps should be minimized. When use of sharps is necessary, they should be disposed of and decontaminated appropriately. All contaminated materials should be effectively decontaminated before removal from the laboratory. If transport to an autoclave is necessary, materials should be double-bagged.”
		Research	“Several species including *R. montanensis*, *R. rhipicephali*, *R. bellii*, *R. amblyommatis*, and *R. canadensis* are not known to cause human disease and may be handled under BSL-2 conditions. New species are frequently described and should be evaluated for appropriate containment on a case-by-case basis.”	As per clinical activity
Canada	PSDS [10]	RMSF		“Containment Level 3 facilities, equipment, and operational practices for work involving infected or potentially infected material, including necropsy of infected animals, arthropods, inoculation, incubation and harvesting of embryonated eggs or tissue cultures. Personnel entering the laboratory should remove street clothing and jewellery, and change into dedicated laboratory clothing and shoes, or don full coverage protective clothing (i.e., completely covering all street clothing). Additional protection may be worn over laboratory clothing when infectious materials are directly handled, such as solid-front gowns with tight fitting wrists, gloves, and respiratory protection. Eye protection must be used where there is a known or potential risk of exposure to splashes. All activities with infectious material should be conducted in a biological safety cabinet (BSC) or other appropriate primary containment device in combination with personal protective equipment. Centrifugation of infected materials must be carried out in closed containers placed in sealed safety cups, or in rotors that are loaded or unloaded in a biological safety cabinet. The use of needles, syringes, and other sharp objects should be strictly limited. Open wounds, cuts, scratches, and grazes should be covered with waterproof dressings. Additional precautions should be considered with work involving animals or large-scale activities”
UK	ACDP [37]	Clinical	“There may be other circumstances or types of work involving biological agents not specified in the list or Annex 1 where full containment measures may not be appropriate. A specific example is work where, although there is a strong indication or likelihood that certain Hazard Group 3 agents might be present, the work will not lead to an increase in the risk of exposure to the agent. For example, blood-borne viruses (BBVs) are unlikely to infect by an airborne route during diagnostic procedures not involving propagation or concentration of the virus (eg haematology), testing of blood donations or transfusion, serology and drug assays. Providing appropriate precautions are taken, not all the stated CL3 measures may be required.”	
		Research	As per clinical activity	As per clinical activity
Australia	AS/NZS 2243.3.2010 [38]	Clinical	“The risk group classifications listed in Table 3.1 to 3.7 are appropriate for small-scale laboratory operations with microorganisms of Risk Groups 2 and 3”	“Where larger volumes or very high concentrations of the microorganisms are to be handled, the risk of infection or inadvertent release from containment can be higher and additional precautions or an increase in physical containment level may be appropriate”
		Research	As per clinical activity	As per research activity
Thailand	Pathogens and Animals Toxins Act 2015 [41]	Clinical	Routine diagnostic laboratory processing within hospital laboratories can be performed in BSL-2 laboratories with strict adherence to Good Laboratory Practice guidelines.	Culturing with the aim of producing large amounts of bacteria should be performed in BSL3 laboratories or equivalent safety level (BSL2 enhanced)
		Research	As per clinical activity	As per research activity

BSL - Biological Safety Level

BMBL - Biosafety in Microbiological and Biomedical Laboratories

ACDP - Advisory Committee on Dangerous Pathogens

PSDS– Pathogen Safety Data Sheet

RMSF– Rocky Mountain Spotted Fever

CL– Biocontainment Level

AS/NZS - Australian/New Zealand Standards

#### Select Agent requirements

In the US, *R. rickettsii* and *R. prowazekii* are classified as Select Agents under the Code of Federal Regulations (42 CFR Part 73), which defines specific regulations regarding the possession, storage, use, and transfer of pathogens [35]. When Select Agent laboratory studies are funded by a US agency, outside of the US, the foreign entity must meet the Select Agent requirements, which often requires review by US government regulators.

#### Post-exposure prophylaxis

Post-exposure prophylaxis (PEP) following a laboratory or clinical exposure to a rickettsial pathogen is potentially lifesaving, depending on the pathogen and the route of inoculation.

Guidance is provided regarding PEP following a laboratory incident, although it is confined to RMSF exposures. The Canadian guidance [10] specifies 100 mg of doxycycline taken twice daily for 5–7 days and until the patient is afebrile for at least 2–3 days (Table S1). The US guidance provided by CDC/NIH [35] detailed in Table S1 does not specify the anti-rickettsial chemotherapy required; however, it also recommends infrastructure development such as availability of an experienced medical officer, signs and symptoms of disease training, non-punitive, anonymous reporting system for accidents; and the reporting of all febrile illnesses.

#### Laboratory-acquired infections

There are numerous reports of laboratory-acquired infections (LAIs) caused by rickettsial pathogens. RMSF has been acquired through needlesticks [43, 44] (Table S1)and aerosol exposures [45], centrifugation errors, equipment failure, the blending of infected ticks, and direct tick bites when working in the field. Before the introduction of biosafety practices (i.e., biological safety cabinets and PPE) and the use of antibiotics for PEP (i.e., before 1940), there were 63 laboratory-related infections recorded, including 11 deaths [10, 46]. Whilst there are some discrepancies in the absolute numbers, many of these LAIs were summarised by Pike [47], where RMSF (*n* = 13), scrub typhus (*n* = 7) and epidemic typhus (*R. prowazekii*) (*n* = 3) fatalities were described. In the same article, the infections and subsequent deaths [47] of Howard Ricketts and S. J. M. von Prowazek due to infections acquired during research activities were eponymously honoured due to their dedication and sacrifice. *Orientia tsutsugamushi* and *R. typhi* LAIs were often the result of aerosol exposures and needlesticks, often without any risk mitigation for aerosol exposure or animal handling [48]. A study aggregating LAI infections in *O. tsutsugamushi* and *R. typhi* reported that between 1931 and 2000, 25 scrub typhus LAIs caused eight deaths and 35 murine typhus infections with no deaths [48], with the cause being largely aerosol and self-inoculation-related [48]. Notably, all *O. tsutsugamushi* LAIs fatalities occurred exclusively during the pre-antibiotic era [48], demonstrating the effectiveness of the tetracycline group PEP. Following the introduction of antibiotic treatments, later reviews recorded ten reports of LAI *R. typhi* between 1941 and 1995, resulting in 35 laboratory-borne infections and no fatal cases distributed in countries such as the US, UK, South Korea, Malaysia, and Switzerland [48].

#### Disinfection and decontamination

Inactivation of rickettsial pathogens normally relies on the use of chemical disinfectants. However, there is often a lack of clear evidence; several reports and guidance documents recommend using chemicals for the empirical inactivation of rickettsial pathogens. Chemical inactivation includes alcohols (ethanol, isopropanol), aldehydes (formaldehyde, paraformaldehyde, glutaraldehyde), alkalis (sodium hydroxide, ammonium hydroxide), halogens (sodium hypochlorite, iodine), peroxygens (accelerated hydrogen peroxide, e.g., Rescue®), potassium peroxymonosulfate (Virkon-S®), peroxyacetic acid, (Oxy-Sept® 333), phenolic compounds (Lysol®, O-Syl®, Amphyl®, TekTrol®, Pheno-Tek II®), sodium dodecyl sulphate (Qiagen ATL); however, specific concentrations and durations of exposure vary and in many cases have not been validated (Table S1). Limited studies have been conducted to evaluate the efficacy of irradiation for inactivating rickettsiae; however, some methodologies are reportedly successful (Table S1). Combinations of chemicals and radiation have been used to inactivate *O. tsutsugamushi;* however, the justification for this combination was mandated by the requirement for rickettsial inactivation in blood products. Thermal treatment is effective for inactivating rickettsial pathogens. Exposure to dry heat at a temperature of 56 °C (Table S1) or humid heat at 121 °C was effective in inactivating most rickettsial pathogens, although there are variations in contact time depending on the pathogen (Table S1).

## Discussion

The review has identified knowledge gaps related to biosafety issues concerning *Orientia* spp. and *Rickettsia* spp. pathogens. A summary of the evidence is presented in Table S1, and an overview of these gaps is presented in Table 4.

**Table 4:** Summary of gaps in biosafety evidence for *Rickettsia* spp. and *Orientia* spp

Biosafety knowledge gap	Description of the issue
Infectious dose	The infectious dose of *Rickettsia* and *Orientia* spp. is considered low but has not been determined.
Classification of risk groups	Debate regarding the risk group classification of *O. tsutsugamushi* - Blacksell et al. [19] advocated for the reclassification of *Orientia* spp. to RG2 from RG3 based on the fact that pathogens in RG2 pose a moderate risk to the individual but a low risk to the community. Furthermore, there is effective treatment available. - RG impacts the practical application of risk, and mitigations, including biosafety levels. The application of risk-based biosafety that considers pathogen and the activity being performed rather than a “one size fits all” approach to risk mitigation and control strategies.
Disinfection & inactivation of *Rickettsia* and *Orientia* spp.	Clear and validated evidence for *Rickettsia* and *Orientia* spp. disinfection & inactivation is patchy or non-existent - Evidence regarding the inactivation of *Rickettsia spp.* by irradiation is limited. - No specific evidence, validation or guidance for o heat and irradiation for *Orientia* spp. have not been determined. o common disinfectants such as ethanol or bleach. o commercial peroxygen disinfectants (i.e., Virkon^®^, Rescue^®^, etc.). o effectiveness and optimal conditions for gaseous decontamination of *Rickettsia* and *Orientia* spp.

### Differentiation of Orientia spp. and Rickettsia spp. biosafety aspects

The evidence presented in this review mainly focuses on *Rickettsia* spp., primarily RMSF and epidemic typhus, which are causes of serious clinical illness. Biosafety and biocontainment measures have been recommended for *Orientia* spp. based on the characteristics of *Rickettsia* spp. However, it is not appropriate to extrapolate similarities between the two organisms regarding susceptibility to physical and chemical inactivation procedures. Such an approach is not comparing “like with like”. Therefore, a “one size fits all” approach is used for *Orientia* spp. biosafety practices are not supported by clear evidence and bypass the principles of risk-based biosafety.

### Inconsistencies in the classification of risk groups

Inconsistencies in applying risk group classifications for *Orientia* spp. and *Rickettsia* spp. have been observed across different jurisdictions, as evidenced in Table 1. The classification of *O. tsutsugamushi* has been subject to debate, which has implications for the practical application of risk and mitigations, including biosafety levels. Implementing risk-based biosafety, which considers both the pathogen being manipulated and the activity being performed, is strongly advocated [1, 49]. It has been proposed that *Orientia* spp. be reclassified as RG2 [19], given that pathogens in this group present a moderate risk to the individual but a low risk to the community, especially given that effective post-exposure prophylaxis is available in the case of laboratory exposures. While there is no argument that *R. rickettsii* and *R. prowazekii* should remain at RG3, we propose that consideration should be given to the reclassification of numerous non-RMSF SFG, TRG, *R. typhi* and *Orientia* spp. to RG2 pathogens.

### Inconsistent recommendations for risk-based biosafety and containment levels

Using a one-size-fits-all approach for in vitro growth of *Orientia* spp. and *Rickettsia* spp. pathogens, such as mandatory BSL3-type containment facilities, inhibits research and does not apply a risk-based approach. The lack of appropriate containment facilities for the culture of rickettsial pathogens was recently recognised in a review of rickettsial diseases in India [50] and Europe [51]. Using a liberal interpretation of the somewhat inconsistent US regulations, implementing BSL3 practices in BSL2 containment laboratory facilities is likely to be sufficient to maintain a safe workplace depending on the scale of the activities with only high-risk pathogens (i.e., *R. rickettsii* and *R. prowazekii*) or large-scale propagation requiring BSL3 practices and containment facilities.

The WHO LBM4 [1] espouses a biosafety approach that comprehensively evaluates risk, enabling customized control measures for laboratory procedures. Blacksell et al. [19] proposed a three-tiered risk classification system (low, medium, and high), facilitating a sustainable and secure work environment while optimizing limited resources. They recommended that low-risk activities involving *Orientia* spp. (i.e., NAATs and serology using clinical specimens) be conducted inside a biological safety cabinet within a BSL2 containment laboratory while employing standard personal protective equipment [19]. This is crucial because most rickettsial illnesses, particularly scrub typhus, manifest in low-resource settings with limited access to high containment facilities. It is possible to safely isolate representative pathogens in small to medium-scale (i.e., low to medium-risk) in vitro experiments to characterize and produce diagnostic reagents. This can be achieved by implementing BSL3 practices in a BSL2 containment laboratory, eliminating the need for high containment facilities and enabling investment in good laboratory practices and procedures for staff. Large-scale culture of *Orientia* spp. and *Rickettsia* spp. would be considered high-risk and performed using BSL3 practices and containment laboratory facilities [19].

### Infectious dose

The infectious dose associated with individual pathogens may differ considerably and contribute significantly to the risk profile and the associated hazards (i.e., low infectious dose = high infectious risk). Accurate determination of the infectious dose for *Rickettsia* and *Orientia* spp. would provide a better understanding of the relative risk-associated strains and inform the accurate determination of pathogen risk groups; however, the existing infectious dose information does provide general guidance. Without a complete understanding of the infectious dose required to cause human infections, or that of surrogate animal models, via various routes, it is impossible to accurately determine the true risks associated with manipulating the pathogen [52–54].

### Disinfection and decontamination

The majority of rickettsial pathogens are considered labile microorganisms [55]. *Rickettsia* spp. can synthesize lipopolysaccharide (LPS), whereas *O. tsutsugamushi* lacks this ability [56]. *Rickettsia* spp. can synthesise a complete peptidoglycan cell wall; however, *O. tsutsugamushi* only possesses a subset of peptidoglycan biosynthesis genes, which generates a less robust peptidoglycan-like structure [56, 57], making it more susceptible to inactivation. The cell membrane of *Orientia* spp. has characteristics that make it more susceptible to chemical and physical inactivation methods than other bacteria classified at lower-risk group levels. There is insufficient evidence to determine the most effective decontamination methods for *Rickettsia* and *Orientia* spp. Claims lack clear supporting evidence and recommendations for chemical disinfectants often lack specific working concentrations and contact times.Validation methods often do not state the pathogen concentration, which can influence the chemical concentration or contact time required to kill the pathogen completely. Peroxygen-based disinfectants (Virkon®, Rescue®, etc.) are widely used in clinical and research labs to inactivate *Rickettsia* and *Orientia* spp. lack evidence. Evidence for inactivating *Rickettsia* spp. by irradiation is limited. Optimal heat and irradiation conditions for *Orientia* spp. are unknown. No evidence exists for gaseous decontamination of *Rickettsia* and *Orientia* spp.

## Conclusions

*Rickettsia* and *Orientia* spp. are neglected diseases, as demonstrated by the lack of evidence-based and specific biosafety information about these pathogens. In the case of *Orientia* spp., most of the available information is derived from *Rickettsia* spp., which may not be appropriate and overstate the risks of working with this pathogen. The advent of effective antibiotic therapy and a better understanding of the true hazards and risks associated with pathogen manipulation should inform decisions, allowing a sustainable and safe work environment.

### Electronic supplementary material

Below is the link to the electronic supplementary material.

Supplementary Material 1. Detailed pathogen biosafety evidence for Rickettsia spp. and Orientia spp.

## Data Availability

No datasets were generated or analysed during the current study.
