# Supplementary material for: Gaps and inconsistencies in the current knowledge and implementation of biosafety and biosecurity practices for rickettsial pathogens
Source: BMC Infect Dis. 2024 Feb 29;24:268. doi: 10.1186/s12879-024-09151-0 (PMC10905923; doi:10.1186/s12879-024-09151-0)
Supplement: Supplementary file 1 — Supplementary Material 1. Detailed pathogen biosafety evidence for Rickettsia spp. and Orientia spp. [file 12879_2024_9151_MOESM1_ESM.docx]

Table S1. Detailed pathogen biosafety evidence for *Rickettsia* spp. and *Orientia* spp.

| Hazards | Hazard classification | Pathogens/Disease or Parameters | Evidence (direct quote) | Reference | Evidence gap (yes/no) |
| --- | --- | --- | --- | --- | --- |
| Modes of Transmission | Arthropod to human | Generic  Generic  *R. typhi* | “Rickettsioses are zoonoses that, except for Q fever, are usually transmitted to humans by arthropods (tick, mite, flea, louse, or chigger).”  “Most rickettsial organisms are transmitted by the bites or infectious fluids (such as feces) inoculated into the skins from ectoparasites such as fleas, lice, mites, and ticks.”  “Flea-borne typhus is spread to people through contact with infected fleas.” | [1]  [2]  [3] | No |
|  | Contact with contaminated blood and needles | *R. rickettsii/*RMSF  *R. rickettsii/*RMSF  *R. conorii* | “Circumstantial evidence indicated that he had acquired his infection through an accidental puncture with a needle from a patient with a fatal illness characterized by rash, headache, myalgia, fever and eventual vascular collapse.” “The diagnosis of Rocky Mountain spotted fever in the physician was confirmed by the serologic results”  “He was a nurse from the hospital where case-patient A was admitted. He reported an accidental percutaneous needlestick injury to his left thumb on July 23, after working with case-patient A in the hospital.” “We confirmed RMSF by using conventional heminested PCR protocol to amplify a 532-bp fragment of the rickettsial ompA gene, as previously described. Rickettsial DNA from the samples generated sequences with 100% identity to the corresponding ompA gene fragment of *R. rickettsii* (GenBank accession no. CP003305).”  “However, cultures from all five LR-WBs at 1 dpi became positive two weeks later (day 14), showing that a certain amount of viable RC successfully passed through the filter. Viable RCs were detectable in all five WB units until 21 dpi and in all five LR-WB units until 7 dpi; three units of both WB and LR-WB had viable RC until 35 dpi.” | [4]  [5]  [6] | No |
|  | Ticks | *Rickettsia* spp.  *Rickettsia* spp.  *Rickettsia* spp. | “In ticks, the transovarial infection rate with different *Rickettsia* spp. ranged from 8 to 100%, while its filial infection rate for the same ranged from 22.7 to 100%”  “All individual adult ticks and larval/nymphal tick pools were PCR-positive for *Rickettsia* spp. Subsequent PCR testing of paired eggs resulted in 91% (30/33) PCR positive among tick egg pools for *Rickettsia* spp.”  “…while 341/1500 (22.7%, 95% CI: 20.6–24.9%) larvae  from 39/50 nests (78.0%, 95% CI: 64.0–88.5%) contained *Rickettsia* spp. DNA” | [7]  [8]  [9] | No |
| Infectious dose | Inoculation | *R. rickettsii*  *R. prowazekii,* *R. typhi R. canada* (*canadensis*), *R. rickettsii, R. conorii, R. sibirica*  *R. rickettsii* | “The average 50% infectious dose among (ID₅₀) exposed human population, N₅₀, is 23 organisms with 95% confidence limits of 1 to 89 organisms.”  “The strain of *R. rickettsii* (ISF126) was consistently more infective than was the Sheila Smith strain (SF116) in all assay systems except that of the mouse, in which interpretation of *Coxiella burnetii* antibody conversion, due to the size of the antibody conversion due to the antigenic mass represented by the inoculum, in both cases was uncertain. It is clear that both *R. conorii* and *R. sibirica* are as infective as the R strain of *R. rickettsii* in the primary CE and L-cell systems. *R. conorii* was the most infective member of the spotted fever group for mice and *R. sibirica* was the next most infective, thus clearly differentiating these two species from *R. rickettsii.”*  “Dogs were rickettsemic for 10 to 14 days after infection. In most animals the level of rickettsemia was ⩾102.5 guinea pig intraperitoneal 50% infectious doses (GPID50).” | [10]  [11]  [12] | No |
| Laboratory acquired infections |  | *R. rickettsii/*RMSF  *R. rickettsii/*RMSF  *R. rickettsii/*RMSF  *R. typhi*/Murine typhus  *Rickettsia prowazekii/*  Epidemic typhus  *O. tsutsugamushi/*  Scrub typhus  *O. tsutsugamushi/*  Scrub typhus  *O. tsutsugamushi/*  Scrub typhus  *R. australis, R. conorii,* and *R. japonica*  *R. akari* | “63 laboratory-acquired infections have been reported as of date with 11 deaths”  “One of the most lethal infections associated with laboratory work is RMSF. All but three of the 13 fatalities occurred in the U.S. T. B. McLintic, A. H. McCray, W. E. Goettinger, and G. H. Cowan, studying the disease in Montana, acquired fatal infections before a vaccine was introduced in 1925, and A. L. Kerlee, who had been vaccinated, died there in 1938. Parker referred to 100% mortality among eight nonvaccinated laboratory workers in Montana, but he did not identify them. The list of RMSF fatalities also includes Lemos Monteira and his assistant, Edison Dias in Brazil, and Breinl, Director of the Institute of Hygiene in Prague. All of the above died before 1940. Subsequently, no fatalities were recorded until 1977 when a laboratory helper and a custodian in Atlanta, Georgia, who had not been immunized, died of RMSF. Their infections appeared to be related to their employment.”  Since 1940, only two laboratory fatalities have occurred due to *R. rickettsii*.  “There have been documented LAIs with murine typhus in 35 individuals contained in 10 reports from 1941 to 1995.”  “Two of the persons who lost their lives while studying epidemic typhus are commemorated in the name *Rickettsia prowazekii*. H. T. Ricketts was apparently infected when one of the lice he collected from typhus patients in Mexico in 1910 escaped from an envelope in his pocket on the way to the laboratory. Studying typhus in Serbia, S. J. M. von Prowazek died in 1915 A. W. Bacot, an Englishman working on typhus in Egypt, acquired a fatal infection in 1922, probably from infected louse excreta.”  “Scrub typhus LAIs were documented in 25 individuals, identified in 11 reports from the period 1931–2000 (Tables 1 and 2). In these reports, there were 8 (32%) deaths and these were all in the preantibiotic era (pre-1950s). The primary routes of infection were cutaneous (accidental self-inoculation and animal bites, 24%) and aerosols (16%), but another 60% were unstated.”  “The first fatal case of laboratory-acquired scrub typhus was reported in Japan in 1931. A technician was infected by the slip of a needle while inoculating a rabbit. About the same time another accident with a needle and syringe, or possibly the same accident, resulted in the death of a research assistant. Phillip memoralized five persons, Dora Lush, P. L. Jones, R. G. Henderson, D. J. Hein, and J. E. Roberts, who died of scrub typhus during World War II. The first four became infected as a result of laboratory accidents, whereas Roberts had performed a necropsy on a scrub typhus patient. An additional fatality may have resulted from a respiratory infection acquired while working with yolk sac material.”  “In the 1980s, the laboratory had three LAI cases from skin wounds caused by the manipulation of glass tubes broken after centrifugation of an infectious suspension. The involved biological agents were Rickettsia species (including *R. australis, R. conorii, and R. japonica*). The persons exposed received appropriate antibiotic treatment and recovered without sequelae.  “A more recent example is the disease called rickettsial pox, caused by *Rickettsia akari*. Shortly after its discovery in New York City in 1946 there were four laboratory-acquired cases of the disease.” | [13]  [14]  [15]  [16]  [14]  [16]  [14]  [17]  [18] | No |
| Disinfection  /Decontamination | Chemical | 1% sodium hypochlorite,  70% ethanol,  2% glutaraldehyde, formaldehyde and phenol  *R. rickettsii* and *R. akari* (generic advice without evidence)  1- 5% phenol dilutions  Lysol, Pine-Sol, Amphyl, O-Syl, Tergisyl, Vesphene, and LpHse  (*Rickettsia* spp. not mentioned – generic advice without evidence)  0.1% formalin - 10 min, Qiagen AVL buffer - 5 min  0.125% β-propiolactone - 1 h  0.0125% β-propiolactone - overnight.  *R. honei*  0.5% phenol - 12 hours  0.1% formaldehyde -24 hours  *R. honei*  1% sodium hypochlorite  *R. prowazekii*  0.1% formalin  *Orientia tsutsugamushi* Karp strain  0.5% formalin  *R. prowazekii*  0.1% Chloramine | “Rickettsia (i.e., *R. rickettsii* and *R. akari*) are expected to be susceptible to 1% sodium hypochlorite, 70% ethanol, 2% glutaraldehyde, formaldehyde and phenol.”  “Phenolics are phenol (carbolic acid) derivatives and typically used at 1- 5% dilutions. These biocides act through membrane damage and are effective against enveloped viruses, rickettsiae, fungi and vegetative bacteria. They also retain more activity in the presence of organic material than other disinfectants. Cresols, hexachlorophene, alkyl- and chloro derivatives and diphenyls are more active than phenol itself. Available commercial products include Lysol, Pine-Sol, Amphyl, O-Syl, Tergisyl, Vesphene, and LpHse.”  “The inactivation limits for rickettsiae were 0.1% formalin about 10 min, Qiagen AVL buffer about 5 min, 56 °C about 5 min, 0.125% β-propiolactone about 1 h, and 0.0125% β-propiolactone overnight.”  “…treatment with 0.5% phenol solution at 30 degrees C for 12 hours and with 0.1% formaldehyde solution at 4 degrees C for 24 hours have been selected.”  “*R. prowazekii* is inactivated by 1% sodium hypochlorite, but no reference to its tolerance to hypochlorite under usual conditions of drinking water disinfection was recovered.”  “A double-strength solution of formalin and merthiolate in diluent was added to freshly prepared yolk sac suspensions, achieving final concentrations of 10% infected yolksac, 0.1% formalin, and 0.01% Merthiolate.”  “A 0.5% solution kills *R. prowazekii* and *R. mooseri* and the rickettsiae of Wolhynan fever in 30 min”  “A 0.5% solution inactivates *R. prowazekii* in 2 min” | [19]  [20]  [21]  [22]  [23]  [24]  [25, 26]  [25, 26] | Possible – Inadequate concentration, and contact time information for many common chemical disinfectants |
|  | Combined  chemicals and radiation | AMT - 0.86 µmol per L combined with UVA light of 5 J per cm.  *O. tsutsugamushi*  50 µmol per L riboflavin and 5.9 J per cm^2^ light.  *O. tsutsugamushi* | “AMT, at 0.86 micromol per L or more, combined with UVA light of 5 J per cm (2), inactivated *O. tsutsugamushi* that contaminated PCs. The PCs that did not receive the combined treatment caused infection.”  “In this study, riboflavin and light were effective in reducing 5 logs of O. tsutsugamushi in blood products, as assayed in an animal model. In RBCs, *O. tsutsugamushi* was inactivated to below the limit of detection by treatment with 500 mmol per L riboflavin and 60 J per cm^2^ light. In PLTs and in plasma, *O. tsutsugamushi* was inactivated to below the limit of detection by treatment with 50 mmol per L riboflavin and 5.9 J per cm^2^ light. Mice injected with riboflavin and light–treated RBCs, PLTs, or treated plasma did not experience any signs or symptoms of infection, 17 days after inoculation.” | [27]  [28] | Yes – lack of specific information relating to inactivation of *Orientia tsutsugamushi* |
|  | Thermal and autoclaving | Moist heat 121°C - 15 min, Dry heat 160 -170°C - 1 hour. *R. akari*  56°C - 5 min *R.honei*  50°C - 1 hour  *R. prowzekii*  56°C - 5 min  *O. tsutsugamushi* | “Rickettsiae can be destroyed by moist heat of 121°C for a minimum of 15 min, or dry heat of 160 -170°C for an hour. *R. akari* is reported to be inactivated rapidly at 56°C.”  “Heating to 56°C about 30 min led to an inactivating effect on rickettsiae for the complete 10^6^ TCIDs. A time kinetic of inactivation showed the inactivating effect al- ready after 5 min”  “As conditions for complete inactivation, incubation at 50 degrees C for 1 hour without chemical disinfectants…”  “Cell suspensions, which were incubated at 56°C for five minutes failed to become infected upon the addition of standard rickettsial inoculum” | [19]  [21]  [22]  [29] | No. |
|  | Radiation | Gamma 200 krads  *Orientia tsutsugamushi* Karp strain | “Mouse lethality was abolished at doses greater than 200 krads…” | [24] | Yes – lack of specific information relating to *Rickettsia* spp. |

Rocky Mountain Spotted Fever (RMSF); 4'-(aminomethyl)-4,5',8-trimethylpsoralen hydrochloride (AMT)

1. DH W: **Chapter 38, Rickettsiae**. *Medical Microbiology 4th edition* 1996.

2. **Rickettsial Diseases-CDC Yellow Book 2024** [<https://wwwnc.cdc.gov/travel/yellowbook/2024/infections-diseases/rickettsial-diseases>]

3. **Flea-borne (murine) typhus** [<https://www.cdc.gov/typhus/murine/index.html>]

4. Sexton DJ, Gallis HA, McRae JR, Cate TR: **Letter: Possible needle-associated Rocky Mountain spotted fever**. *N Engl J Med* 1975, **292**(12):645.

5. Vilges de Oliveira S, Faccini-Martínez ÁA, Adelino TER, de Lima Duré AÍ, Barbieri ARM, Labruna MB: **Needlestick-Associated Rocky Mountain Spotted Fever, Brazil**. *Emerging infectious diseases* 2020, **26**(4):815-816.

6. Lucchese L, Ravagnan S, Da Rold G, Toniolo F, Wurzburger W, Mion M, Carminato A, Fournier P-E, Capelli G, Natale A *et al*: **Survival of *Rickettsia conorii* in artificially contaminated whole and leukoreduced canine blood units during the storage period**. *Parasites & Vectors* 2020, **13**(1):118.

7. Ravindran R, Hembram PK, Kumar GS, Kumar KGA, Deepa CK, Varghese A: **Transovarial transmission of pathogenic protozoa and rickettsial organisms in ticks**. *Parasitology Research* 2023, **122**(3):691-704.

8. Moore TC, Pulscher LA, Caddell L, von Fricken ME, Anderson BD, Gonchigoo B, Gray GC: **Evidence for transovarial transmission of tick-borne rickettsiae circulating in Northern Mongolia**. *PLoS Negl Trop Dis* 2018, **12**(8):e0006696.

9. Hauck D, Jordan D, Springer A, Schunack B, Pachnicke S, Fingerle V, Strube C: **Transovarial transmission of *Borrelia* spp., *Rickettsia* spp. and *Anaplasma phagocytophilum* in *Ixodes ricinus* under field conditions extrapolated from DNA detection in questing larvae**. *Parasit Vectors* 2020, **13**(1):176.

10. Tamrakar SB, Haas CN: **Dose-response model of Rocky Mountain spotted fever (RMSF) for human**. *Risk Anal* 2011, **31**(10):1610-1621.

11. Ormsbee R, Peacock M, Gerloff R, Tallent G, Wike D: **Limits of rickettsial infectivity**. *Infect Immun* 1978, **19**(1):239-245.

12. Keenan KP, Ruhles WC, Jr, Huxsoll DL, Williams RG, Hildebrandt PK, Campbell JM, Stephenson EH: **Pathogenesis of Infection with *Rickettsia rickettsii i*n the Dog: A Disease Model for Rocky Mountain Spotted Fever**. *The Journal of Infectious Diseases* 1977, **135**(6):911-917.

13. **Pathogen Safety Data Sheets: Infectious Substances – *Rickettsia rickettsii*** [<https://www.canada.ca/en/public-health/services/laboratory-biosafety-biosecurity/pathogen-safety-data-sheets-risk-assessment/rickettsia-rickettsii.html>]

14. Pike RM: **Laboratory-associated infections: incidence, fatalities, causes, and prevention**. *Annu Rev Microbiol* 1979, **33**:41-66.

15. Centers for Disease Control and Prevention. National Institutes of Health: **Biosafety in Microbiological and Biomedical Laboratories**, vol. 2023, 6th edn; 2020.

16. Blacksell SD, Robinson MT, Newton PN, Day NPJ: **Laboratory-acquired Scrub Typhus and Murine Typhus Infections: The Argument for a Risk-based Approach to Biosafety Requirements for Orientia tsutsugamushi and Rickettsia typhi Laboratory Activities**. *Clinical infectious diseases : an official publication of the Infectious Diseases Society of America* 2019, **68**(8):1413-1419.

17. Wurtz N, Papa A, Hukic M, Di Caro A, Leparc-Goffart I, Leroy E, Landini MP, Sekeyova Z, Dumler JS, Bădescu D *et al*: **Survey of laboratory-acquired infections around the world in biosafety level 3 and 4 laboratories**. *European Journal of Clinical Microbiology & Infectious Diseases* 2016, **35**(8):1247-1258.

18. **Microbiological safety in US and foreign laboratories** [<https://apps.dtic.mil/sti/pdfs/AD0268635.pdf>]

19. **Spotted Fevers (including Rocky Mountain Spotted Fever and Mediterranean Spotted Fever)** [<https://www.cfsph.iastate.edu/Factsheets/pdfs/RMSF.pdf>]

20. **Fact sheet Chemical disinfectants** [<https://ibc.utah.edu/_resources/documents/fact-sheets-and-sops/chemical-disinfectants_fact-sheet.pdf>]

21. Frickmann H, Dobler G: **Inactivation of rickettsiae**. *European journal of microbiology & immunology* 2013, **3**(3):188-193.

22. Eremeeva ME, Ignatovich VF, Popov VL, Balaeva NM: **Inactivation of concentrated biomasses of *Rickettsia prowazekii***. *Zh Mikrobiol Epidemiol Immunobiol* 1989(7):12-17.

23. W Dickinson Burrows SER: **Biological Warfare Agents as Threats to Potable Water**. *US Army Center for Health Promotion and Preventive Medicine* 1999.

24. Eisenberg GH, Jr., Osterman JV: **Experimental scrub typhus immunogens: gamma-irradiated and formalinized rickettsiae**. *Infect Immun* 1977, **15**(1):124-131.

25. Bereznsyukova VA: **Resistance of the rickettsiae of typhus fever, murine typhus, and Wolhynian fever to disinfectants and insecticides.**: Molotov; 1952.

26. Zdrodovskii PF, Golinevich HM: **The Rickettsial Diseases** New York and Oxford: Pergamon Press; 1960.

27. Belanger KJ, Kelly DJ, Mettille FC, Hanson CV, Lippert LE: **Psoralen photochemical inactivation of *Orientia tsutsugamushi* in platelet concentrates**. *Transfusion* 2000, **40**(12):1503-1507.

28. Rentas F, Harman R, Gomez C, Salata J, Childs J, Silva T, Lippert L, Montgomery J, Richards A, Chan C *et al*: **Inactivation of *Orientia tsutsugamushi* in red blood cells, plasma, and platelets with riboflavin and light, as demonstrated in an animal model**. *Transfusion* 2007, **47**(2):240-247.

29. Cohn ZA, Bozeman FM, Campbell JM, Humphries JW, Sawyer TK: **Study on growth of Rickettsia. V. Penetration of Rickettsia tsutsugamushi into mammalian cells in vitro**. *J Exp Med* 1959, **109**(3):271-292.
